# Supplementary material for: Nicotinic Receptor Subunits Atlas in the Adult Human Lung
Source: Int J Mol Sci. 2020 Oct 9;21(20):7446. doi: 10.3390/ijms21207446 (PMC7588933; doi:10.3390/ijms21207446)

International Journal of *Molecular Sciences*

MDPI

Supporting information

Nicotinic receptor subunits atlas in the adult human lung

Zania Diabasana, Jeanne-Marie Perotin[1], Randa Belgacemi, Julien Ancel, Pauline Mulette, Gonzague Delepine, Philippe Gosset, Uwe Maskos, Myriam Polette, Gaëtan Deslée and Valérian Dormoy

1. Supporting information Tables

Table S1. Percentages of detection of each nAChR subunit in whole lung tissue transcriptomes.

	Non-Smokers (n=42)		Smokers (n=111)		
CHRN	Detection (%)	SD	Detection (%)	SD	p
A1	31.11	11.48	40.17	15.96	***
A2	29.43	18.56	42.70	15.97	***
A3	19.64	35.77	4.65	19.64	*
A4	21.93	39.78	5.04	21.21	*
A5	45.39	27.60	47.80	15.67	ns
A6	50.95	30.07	57.04	16.94	ns
A7	0.00	0.0	25.53	34.62	NA
A9	55.71	19.48	31.49	26.00	***
A10	71.02	17.65	68.19	15.47	ns
B1	80.99	11.40	81.80	9.33	ns
B2	20.37	37.01	4.61	19.40	*
B3	53.87	31.47	64.87	18.65	*
B4	26.84	18.74	46.92	26.15	***
D	65.00	13.14	40.45	31.39	***
E	76.07	13.68	75.21	8.35	ns
G	21.05	38.22	4.73	19.88	*

ns, non-significate; SD, standard deviation ; NA, not applicable. Coloured subunits indicate upregulation (green) and downregulation (red) in both groups when statistically significate.

Table S2. Repartitions of the gene expressions of each nAChR subunit in whole lung tissue transcriptomes.

	Non-Smokers (n=42)		Smokers (n=111)		
CHRN	Expression (%)	SD	Expression (%)	SD	p
A1	7.09	2.36	9.20	3.44	***
A2	4.20	2.59	6.32	2.37	***
A3	2.17	3.94	0.51	2.14	***
A4	2.05	3.72	0.47	1.97	***
A5	7.00	4.17	7.59	2.40	ns
A6	6.38	3.76	7.31	2.06	ns

**A7**	0.00	0.00	2.80	3.81	NA
**A9**	5.13	2.12	2.84	2.42	***
**A10**	14.79	3.23	14.34	3.00	ns
**B1**	15.29	1.72	15.55	1.618	ns
**B2**	2.66	4.83	0.60	2.51	***
**B3**	6.33	3.68	7.83	2.17	**
**B4**	3.47	2.35	6.33	3.64	***
**D**	8.15	1.79	5.02	3.91	***
**E**	12.76	1.85	12.73	1.22	ns
**G**	2.53	4.58	0.56	2.37	***

ns, non-significate; SD, standard deviation ; NA, not applicable. Coloured subunits indicate upregulation (green) and downregulation (red) in both groups when statistically significate.

Table S3. Percentages of detection of each nAChR subunit in LAEC transcriptomes.

	Non-Smokers (n=5)		Smokers (n=5)		
CHRN	Detection (%)	SD	Detection (%)	SD	p
**A1**	0.00	0.00	0.00	0.00	NA
**A2**	0.00	0.00	0.00	0.00	NA
**A3**	20.00	44.72	6.48	14.50	ns
**A4**	0.00	0.00	0.00	0.00	NA
**A5**	79.80	33.01	45.57	15.82	*
**A6**	20.00	44.72	13.57	30.33	ns
**A7**	74.13	23.32	47.17	30.96	ns
**A9**	49.00	35.99	32.56	28.86	ns
**A10**	59.49	25.23	28.97	18.51	*
**B1**	0.00	0.00	34.44	48.17	NA
**B2**	8.88	19.88	28.50	44.00	ns
**B3**	0.00	0.00	0.00	0.00	NA
**B4**	0.00	0.00	20.00	44.72	NA
**D**	0.00	0.00	0.00	0.00	NA
**E**	70.50	23.07	39.02	15.03	*
**G**	13.71	30.65	25.86	43.34	ns

ns, non-significate; SD, standard deviation ; NA, not applicable. Coloured subunits indicate upregulation (green) and downregulation (red) in both groups when statistically significate.

Table S4. Repartitions of the gene expressions of each nAChR subunit in LAEC transcriptomes.

	Non-Smokers (n=5)		Smokers (n=5)		
CHRN	Expression (%)	SD	Expression (%)	SD	p
**A1**	0.00	0.00	0.00	0.00	ns
**A2**	0.00	0.00	0.00	0.00	ns
**A3**	1.01	2.26	0.50	1.13	ns
**A4**	0.00	0.00	0.00	0.00	ns
**A5**	17.98	9.69	19.73	18.14	ns

**A6**	0.73	1.63	0.70	1.56	ns
**A7**	33.29	12.50	24.63	16.38	ns
**A9**	22.82	14.14	20.23	16.04	ns
**A10**	18.00	2.99	11.26	7.32	ns
**B1**	0.00	0.00	1.81	2.63	NA
**B2**	0.60	1.33	11.02	21.25	NA
**B3**	0.00	0.00	0.00	0.00	ns
**B4**	0.00	0.00	0.61	1.37	NA
**D**	0.00	0.00	0.00	0.00	ns
**E**	2.99	3.21	6.38	6.25	ns
**G**	2.58	5.77	3.13	5.15	ns

ns, non-significate; SD, standard deviation ; NA, not applicable.

Table S5. Percentages of detection of each nAChR subunit in SAEC transcriptomes.

	**Non-Smokers (n=63)**		**Smokers (n=72)**		
**CHRN**	**Detection (%)**	**SD**	**Detection (%)**	**SD**	**p**
**A1**	19.76	18.19	17.94	16.64	ns
**A2**	40.37	19.26	38.47	18.26	ns
**A3**	28.09	18.87	29.69	21.35	ns
**A4**	26.91	21.26	26.16	20.95	ns
**A5**	19.34	12.45	25.44	19.05	*
**A6**	44.07	22.46	49.31	21.30	ns
**A7**	33.33	17.59	42.75	23.26	**
**A9**	42.73	22.00	39.41	17.81	ns
**A10**	35.52	17.83	35.41	16.35	ns
**B1**	39.45	21.82	42.64	20.75	ns
**B2**	35.57	17.80	48.21	20.14	***
**B3**	29.03	20.59	37.20	21.75	*
**B4**	15.82	13.51	20.19	20.67	ns
**D**	21.04	18.37	20.74	16.82	ns
**E**	26.68	17.79	32.67	23.12	ns
**G**	29.29	20.95	30.43	23.50	ns

ns, non-significate; SD, standard deviation. Coloured subunits indicate upregulation (green) and downregulation (red) in both groups when statistically significate.

Table S6. Repartitions of the gene expressions of each nAChR subunit in SAEC transcriptomes.

	**Non-Smokers (n=63)**		**Smokers (n=72)**		
**CHRN**	**Expression (%)**	**SD**	**Expression (%)**	**SD**	**p**
**A1**	2.41	1.70	2.03	1.68	ns
**A2**	9.43	3.86	8.13	3.77	*
**A3**	4.23	2.37	3.95	2.46	ns
**A4**	3.37	2.22	3.04	2.33	ns

**A5**	2.83	1.56	3.24	1.81	ns
**A6**	4.95	2.00	5.11	1.99	ns
**A7**	8.61	3.46	9.93	4.61	*
**A9**	13.88	5.53	11.99	4.86	*
**A10**	13.76	5.08	12.62	4.55	ns
**B1**	6.87	3.07	6.78	2.81	ns
**B2**	13.89	4.98	16.81	5.17	**
**B3**	3.41	1.83	4.09	2.00	*
**B4**	2.14	1.35	2.46	2.04	ns
**D**	2.14	1.66	1.86	1.38	ns
**E**	3.41	2.34	3.62	2.57	ns
**G**	4.67	2.65	4.34	2.67	ns

ns, non-significate; SD, standard deviation. Coloured subunits indicate upregulation (green) and downregulation (red) in both groups when statistically significate.

Table S7. List of CHRN antibodies.

Antibodies	Species	Reference	Companies	Concentrations
**α1**	Rabbit	HPA071554	Sigma-Aldrich	1:100
**α2**	Mouse	NBP2-61667	Novus Biological	1:50
**α3**	Rabbit	HPA029430	Sigma-Aldrich	1:100
**α4**	Mouse	NBP2-61674	Novus Biological	1:100
**α5**	Rabbit	HPA054381	Sigma-Aldrich	1:50
**α6**	Mouse	NBP2-61679	Novus Biological	1:100
**α7**	Mouse	NBP2-61738	Novus Biological	1:100
**α9**	Rabbit	26025-1-AP	Proteintech	1:100
**α10**	Mouse	NBP2-61666	Novus Biological	1:50
**β1**	Rabbit	HPA005822	Sigma-Aldrich	1:100
**β2**	Rabbit	17844-1-AP	Proteintech	1:50
**β3**	Rabbit	APrEST84413	Novus Biological	1:100
**β4**	Mouse	NBP2-61742	Novus Biological	1:100
**δ**	Rabbit	HPA056404	Sigma-Aldrich	1:100
**ε**	Rabbit	NBP1-79951	Novus Biological	1:100
**γ**	Rabbit	NBP1-79952	Novus Biological	1:100

Table S8. List of recognition antigens of CHRN antibodies and their percentages of identity.

s.u.	Antigenic sequences	Position	Identity*
**α1**	MKLGTWTYDGSVVAINPESDQPDLSNFMESGEWVIKESRGWKHSVTYSCCPDTPYLDITYHF	189-250	α2/3/4/6
**α2**	EEAKRPPPRAPGDPLSSPSPTALPQGGSHTETEDRLFKHLFRGYNRWARPVPNTSDVVIVRFGLSIAQLIDVDEKNQMMTTNVWLKQEWSDYKLRWNPTDFGNITSLRVPSEMIWIPDIVLYNNADGEFAVTHMTKAHLFSTGTVHWVPPAIYKSSCSIDVTFFPFDQQNCKMKFGSWTYDKAKIDLEQMEQTVDLKDYWESGEWAIVNATGTYNSKKYDCCAEIYPDVTYAFVIRRL	27-264	α4<br>α3/5/6<br>β3
**α3**	RTPTTHTMPSWVKTVFLNLLPRVMFMTRPTSNEGNAQKPRPLYGAELSNLNCFSRAESKGCKEGYPCQDGMCGYCHHRRIKISNFSANLTRSSSSESVDAVLSLSALSPEIKEAIQSVKYIAENMKAQNEAKEIQDDWKYVAM	331-473	α1/2/6<br>β2/4
**α4**	HVETRAHAEERLLKKLFSGYNKWSRPVANISDVVLVRFGLSIAQLIDVDEKNQMMTTNVWVKQEWHDYKLRWDPADYENVTSIRIPSELIWRPDIVLYNNADGDFAVTHLTKAHLFHDGRVQWTPPAIYKSSCSIDVTFFPFDQQNCTMKFGSWTYDKAKIDLVNMHSRVDQLDFWESGEWVIVDAVGTYNTRKYECCAEIYPDITYAFVIRRL	29-242	α2<br>α3/5/6<br>β3
**α5**	AQRGLSEPSSIAKHEDSLLKDLFQDYERWVRPVEHLNDK	33-71	β3<br>α4
**α6**	KGCVGCATEERLFHKLFSHYNQFIRPVENVSDPVTVHFEVAITQLANVDEVNQIMETNLWLRHIWNDYKLRWDPMEYDGIETLRVPADKIWKPDIVLYNNAVGDFQVEGKTKALLKYNGMITWTPPAIFKSSCPMDITFFPFDHQNCSLKFGSWTYDKAEIDLLIIGSKVDMNDFWENSEWEIIDASGYKHDIKYNCCEEIYTDITYSFYIRRL	26-239	α3<br>α2/4/5<br>β3
**α7**	LYKELVKNYNPLERPVANDSQPLTVYFSLSLLQIMDVDEKNQVLTTNIWLQMSWTDHYLQWNVSEYPGVKTVRFPDGQIWKPDILLYNSADE	58-149	α2/3/4/10
**α9**	HFCGAEARPVPHWARVVILKYMSRVLFVYDVGESCLSPHHSRERDHLTKVYSKLPESNLKAARNKDLSRKKDMNKRLKNDLGCQGKNPQEAESYCAQYKVLTRNIEYIAKCLKDHKATNSKGSEWKKVAKVIDRFFMWIFFIMVFVMTILIIARAD	324-479	α2/4/6/10
**α10**	AEGRLALKLFRDLFANYTSALRPVADTDQTLNVTLEVTLSQIIDMDERNQVLTLYLWIRQEWTDAYLRWDPNAYGGLDAIRIPSSLVWRPDIVLYNKADAQPPGSASTNVVLRHDGAVRWDAPAITRSSCRVDVAAFPFDAQHCGLTFGSWTHGGHQLDVRPRGAAASLADFVENVEWRVLGMPARRRVLTYGCCSEPYPDVTFTLLLRRRAA	25-237	α9<br>α3/7
**β1**	LSVVVLNLHHRSPHTHQMPLWVRQIFIHKLPLYLRLKRPKPERDLMPEPPHCSSPGSGWGRGTDEYFIRKPPSDFLFPKPNRFQPELSAPDLRRFIDGPNRAVALLPELREVVSSISYIARQLQEQEDHDALKEDWQF	325-462	α3<br>β2/4
**β2**	LLRLCSGVWGTDTEERLVEHLLDPSRYNKLIRPATNGSELVTVQLMVSLAQLISVHEREQIMTTNVWLTQEWEDYRLTWKPEEFDNMKKVRLPSKHIWLPDVVLYNNADGMYEVSFYSNAVVSYDGSIFWLPPAIYKSACKIEVKHFPFDQQNCTMKFRSWTYDRTEIDLVLKSEVASLDDFTPSGEWDIVALPGRRNENPDDSTYVDITYD	16-227	β4<br>α2

**β3**	TGFNSIAENEDALLRHLFQGYQKWVRPVLHSNDTI	20-54	α5<br>α2/4
**β4**	CRVANAEEKLMDDLLNKTRYNNLIRPATSSSQLISIKLQLSLAQLISVNEREQIMTTNVWLKQ<br>EWTDYRLTWNSSRYEGVNILRIPAKRIWLPDIVLYNNADGTYEVSVYTNLIVRSNGSVLWLP<br>PAIYKSACKIEVKYFPFDQQNCTLKFRSWTYDHTEIDMVLMTPTASMDDFTPSGEWDIVALP<br>GRRTVNPQDPSYVDVTYDFIIKRKPLFYT	21-236	β2<br>α3<br>β3
δ	LVRRSSSLGYISKAEEYFLLKSRSDLMFEKQSERHGLARRLTTARRPPASSEQAQQELFNELKP<br>AVDGANFIVNHMRDQNNYNEEKDSWNR	374-464	β2
ε	GLLGRGVGKNEELRLYHHLFNNYDPGSRPVREPEDTVTISLKVTLTNLIS	13-62	α6, γ
γ	NYDPNLRPAERDSDVVNVSLKLTLTNLISLNEREEALTTNVWIEMQWCDY	36-85	δ<br>α7, β2/4

* Range of the percentage of identity obtained from blastp: yellow, 65-80%; light green, 50-65%; dark green, <50%.

Table S9. List of primers.

ENES	GenBank	Forward sequence	Reverse sequence	Amplicon size (b)
**CHRNA1**	NM_001039523.2	5′- GTCCACACAAGCTCCGGTA-3′	5′- CAGACGGGTCTCATGTTCG-3′	104
**CHRNA2**	NM_000742.3	5′- CTGTGGTGGCTCCTTCTGA-3′	5′- GGGAGAGGAGAGTGGGTCTC-3′	87
**CHRNA3**	NM_000743.4	5′- TGAAATGGAACCCCTCTGAC-3′	5′- GAAATCCCCAACAGCATTGT-3′	107
**CHRNA4**	NM_000744.6	5′- GCCGGACATCGTCCTCTAC-3′	5′- TGCAGGAGCTCTTGTAAATGG-3′	125
**CHRNA5**	NM_000745.3	5′- GACAACAAACGTCTGGTTGAAA-3′	5′- ACAGAGTCTGAAGGAACACGTATAAC-3′	105
**CHRNA6**	NM_004198.3	5′- TTCATGGGGGCTTGTGTC-3′	5′- GAGCCTCTCCTCAGTTGCAC-3′	83
**CHRNA7**	NM_000746.5	5′- CAATGACTCGCAACCACTCA-3′	5′- GTGATCTGTCCAAGACATTTGC-3′	121
**CHRNA9**	NM_017581.3	5′-TCAGAAAATGTGCCCCTGAT-3′	5′- GGCCCCACAGAAGTGGATA-3′	108
**CHRNA10**	NM_020402.3	5′- CCCAGATCATCGACATGGA-3′	5′- CCCATCGTAGGTAGGCATCT-3′	90
**CHRNB1**	NM_000747.2	5′- CACAAAGGTGTACTTAGACCTGGA-3′	5′- TTCAGTAGCACCACGTCAGG-3′	129
**CHRNB2**	NM_000748.2	5′- CTGGCCCAGCTCATCAGT-3′	5′- TCCAGGTGAGGCGATAATCT-3′	94
**CHRNB3**	NM_000749.4	5′- GGTCCGCCCTGTATTACATTC-3′	5′- TCAGCTGATTCTTTTCATCCAC-3′	95
**CHRNB4**	NM_000750.4	5′- TGACGATGAAGACCAGAGTGTC-3′	5′- GGACGCACACAAACATGAAC-3′	95
**CHRND**	NM_000751.3	5′- GGGACCAGAACAATTACAATGAG-3′	5′- GCAGGAAGATCCAGGCTGT-3′	113
**CHRNE**	NM_000080.4	5′- CGACACAGAGGCCTATACTGAG-3′	5′- GCGGATGATGAGCGAGTAG-3′	93
**CHRNG**	NM_005199.4	5′- AGCAGAGTCACTTTGACAATGG-3′	5′- GTAGTGGGCCATGAGGAAGA-3′	131

2. Supporting information Figures

Figure S1. Constraint-based Multiple Alignment of CHRN antibodies. COBALT alignment is shown for the 16 subunits and antigen sequences of corresponding antibodies are highlighted in grey. Red amino acids are conserved for all subunits.

```
α1    001  MEPW--------PLLLL-------FSLCS--------AGL-------VLGSEH------------ETRLVAKLFKD--YS  36
α2    001  MGPS-cpvflSFTKLSLwwllLTPAGGEEakr-ppprAPGdplsspsPTALPQggshte-t----EDRLFKHLFRG--YN  71
α3    001  M----gsgplSLPLALSpprlLLLLLLSL--------LPV-------ARASEA------------EHRLFERLFED--YN  47
α4    001  M----------------------ELGGPGapr-llp-----plllllGTGLLRasshve-trahaEERLLKKLFSG--YN  49
α5    001  MAAR-gsgprALRLLLL---vQLVAGRCG--------LAG-------AAGGAQrglsepssiakhEDSLLKDLFQD--YE  59
α6    001  MLTSkgqgflHGGLCL-----WLCVFTPF--------FKG-------CVGCAT------------EERLFHKLFSH--YN  46
α7    001  MRCSpggvwlALAASLLhg--KATASPPStppwdpghIPG-------ASVRPApgpvsl--qgefQRKLYKELVKN--YN  67
α9    001  ---------------MNwshsCISF-------------CW-------IYFAASrlraaetadgkyAQKLFNDLFED--YS  43
α10   001  ---------------MGlrshHLSLGLLLlfl-lpaeCLG-------AEGRLAl-------------KLFRDLFAN--YT  42
β1    001  MTPG--------ALLMLlg----ALGAPL--------APG-------VRGSEA------------EGRLREKLFSG--YD  39
β2    001  MARRcgpvalLLGFGL--------LRLCS----------G-------VWGTDT------------EERLVEHLLDPsrYN  43
β3    001  M----------LPDFML---vLIVLG---------------------IPSSATtgfn---siaenEDALLRHLFQG--YQ  41
β4    001  MRR--apslvL--FFL--------VALCG--------RGN-------CRVANA------------EEKLMDDLLNKtrYN  41
γ     001  MHGG------QGPLLLL-----LLLAVCL----------G-------AQGRNQ------------EERLLADLMQN--YD  38
ε     001  MARA------PLGVLLLl---------------------G-------LLGRGV-gkne-------ELRLYHHLFNN--YD  36
δ     001  MEGP------VLTLGLL-----AALAVC-----------G-------SWGLNE------------EERLIRHLFQEkgYN  39

α1    037  SVVRPVEDHRQVVEVTVGLQLIQLINVDEVNQIVTTNVRLKQgdmvdlprpscvtlgvplfshlqneQWVDYNLKWNPDD  116
α2    072  RWARPVPNTSDVVIVRFGLSIAQLIDVDEKNQMMTTNVWLKQ-------------------------EWSDYKLRWNPTD  126
α3    048  EIIRPVANVSDPVIIHFEVSMSQLVKVDEVNQIMETNLWLKQ-------------------------IWNDYKLKWNPSD  102
α4    050  KWSRPVANISDVVLVRFGLSIAQLIDVDEKNQMMTTNVWVKQ-------------------------EWHDYKLRWDPAD  104
α5    060  RWVRPVEHLNDKIKIKFGLAISQLVDVDEKNQLMTTNVWLKQ-------------------------EWIDVKLRWNPDD  114
α6    047  QFIRPVENVSDPVTVHFEVAITQLANVDEVNQIMETNLWLRH-------------------------IWNDYKLRWDPME  101
α7    068  PLERPVANDSQPLTVYFSLSLLQIMDVDEKNQVLTTNIWLQM-------------------------SWTDHYLQWNVSE  122
α9    044  NALRPVEDTDKVLNVTLQITLSQIKDMDERNQILTAYLWIRQ-------------------------IWHDAYLTWDRDQ  98
α10   043  SALRPVADTDQTLNVTLEVTLSQIIDMDERNQVLTLYLWIRQ-------------------------EWTDAYLRWDPNA  97
β1    040  SSVRPAREVGDRVRVSVGLILAQLISLNEKDEEMSTKVYLDL-------------------------EWTDYRLSWDPAE  94
β2    044  KLIRPATNGSELVTVQLMVSLAQLISVHEREQIMTTNVWLTQ-------------------------EWEDYRLTWKPEE  98
β3    042  KWVRPVLHSNDTIKVYFGLKISQLVDVDEKNQLMTTNVWLKQ-------------------------EWTDHKLRWNPDD  96
β4    042  NLIRPATSSSQLISIKLQLSLAQLISVNEREQIMTTNVWLKQ-------------------------EWTDYRLTWNSSR  96
γ     039  PNLRPAERDSDVVNVSLKLTLTNLISLNEREEALTTNVWIEM-------------------------QWCDYRLRWDPRD  93
ε     037  PGSRPVREPEDTVTISLKVTLTNLISLNEKEETLTTSVWIGI-------------------------DWQDYRLNYSKDD  91
δ     040  KELRPVAHKEESVDVALALTLSNLISLKEVEETLTTNVWIEH-------------------------GWTDNRLKWNAEE  94

α1    117  YGGVKKIHIPSEKIWRPDLVLYNNADGDFAIVKFTKVLLQYTGHITWTPPAIFKSYCEIIVTHFPFDEQNCSMKLGTWTY  196
α2    127  FGNITSLRVPSEMIWIPDIVLYNNADGEFAVTHMTKAHLFSTGTVHWVPPAIYKSSCSIDVTFFPFDQQNCKMKFGSWTY  206
α3    103  YGGAEFMRVPAQKIWKPDIVLYNNAVGDFQVDDKTKALLKYTGEVTWIPPAIFKSSCKIDVTYFPFDYQNCTMKFGSWSY  182
α4    105  YENVTSIRIPSELIWRPDIVLYNNADGDFAVTHLTKAHLFHDGRVQWTPPAIYKSSCSIDVTFFPFDQQNCTMKFGSWTY  184
α5    115  YGGIKVIRVPSDSVWTPDIVLFDNADGRFEGT-STKTVIRYNGTVTWTPPANYKSSCTIDVTFFPFDLQNCSMKFGSWTY  193
α6    102  YDGIETLRVPADKIWKPDIVLYNNAVGDFQVEGKTKALLKYNGMITWTPPAIFKSSCPMDITFFPFDHQNCSLKFGSWTY  181
α7    123  YPGVKTVRFPDGQIWKPDILLYNSADERFDATFHTNVLVNSSGHCQYLPPGIFKSSCYIDVRWFPFDVQHCKLKFGSWSY  202
α9    99   YDGLDSIRIPSDLVWRPDIVLYNKADDESSEPVNTNVVLRYDGLITWDAPAITKSSCVVDVTYFPFDNQQCNLTFGSWTY  178
α10   98   YGGLDAIRIPSSLVWRPDIVLYNKADAQPPGSASTNVVLRHDGAVRWDAPAITRSSCRVDVAAFPFDAQHCGLTFGSWTH  177
β1    95   HDGIDSLRITAESVWLPDVVLLNNNDGNFDVALDISVVVSSDGSVRWQPPGIYRSSCSIQVTYFPFDWQNCTMVFSSYSY  174
β2    99   FDNMKKVRLPSKHIWLPDVVLYNNADGMYEVSFYSNAVVSYDGSIFWLPPAIYKSACKIEVKHFPFDQQNCTMKFRSWTY  178
β3    97   YGGIHSIKVPSESLWLPDIVLFENADGRFEGSLMTKVIVKSNGTVVWTPPASYKSSCTMDVTFFPFDRQNCSMKFGSWTY  176
β4    97   YEGVNILRIPAKRIWLPDIVLYNNADGTYEVSVYTNLIVRSNGSVLWLPPAIYKSACKIEVKYFPFDQQNCTLKFRSWTY  176
γ     94   YEGLWVLRVPSTMVWRPDIVLENNVDGVFEVALYCNVLVSPDGCIYWLPPAIFRSACSISVTYFPFDWQNCSLIFQSQTY  173
ε     92   FGGIETLRVPSELVWLPEIVLENNIDGQFGVAYDANVLVYEGGSVTWLPPAIYRSVCAVEVTYFPFDWQNCSLIFRSQTY  171
δ     95   FGNISVLRLPPDMVWLPEIVLENNNDGSFQISYSCNVLVYHYGFVYWLPPAIFRSSCPISVTYFPFDWQNCSLKFSSLKY  174

α1    197  DGSVVAINPESDQP------------DLSNFMESGEWVIKESRGWK---HSVTYSCCPDTpYLDITYHFVMQRLPLYFIV  261
α2    207  DKAKIDLEQMEQTV------------DLKDYWESGEWAIVNATGTYNS---KKYDCCAEI-YPDVTYAFVIRRLPLFYTI  270
α3    183  DKAKIDLVLIGSSM------------NLKDYWESGEWAIIKAPGYK---HDIKYNCCEEI-YPDITYSLYIRRLPLFYTI  246
α4    185  DKAKIDLVNMHSRV------------DQLDFWESGEWVIVDAVGTYNT---RKYECCAEI-YPDITYAFVIRRLPLFYTI  248
α5    194  DGSQVDIILEDQDV------------DKRDFFDNGEWEIVSATGSK---GNRTDSCCW---YPYVTYSFVIKRLPLFYTL  255
α6    182  DKAEIDLLIIGSKV------------DMNDFWENSEWEIIDASGYK---HDIKYNCCEEI-YTDITYSFYIRRLPMFYTI  245
α7    203  GGWSLDLQMQEADI--------------SGYIPNGEWDLVGIPGKRSE---RFYECCKEP-YPDVTFTVTMRRRTLYYGL  264
α9    179  NGNQVDIFNALDSG------------DLSDFIEDVEWEVHGMPAVKNV---ISYGCCSEP-YPDVTFTLLLKRRSSFYIV  242
α10   178  GGHQLDVRPRGAAA------------SLADFVENVEWRVLGMPARRRV---LTYGCCSEP-YPDVTFTLLLRRRAAAYVC  241
β1    175  DSSEVSLQTGLGPDgqghq---eihiHEGTFIENGQWEIIHKPSRLIQpPGDPRGGREGQ-RQEVIFYLIIRRKPLFYLV  250
β2    179  DRTEIDLVLKSEVA------------SLDDFTPSGEWDIVALPGRR---NENPDDST----YVDITYDFIIRRKPLFYTI  239
β3    177  DGTMVDLILINENV------------DRKDFFDNGEWEILNAKGMK---GNRRDGVYS---YPFITYSFVLRRLPLFYTL  238
β4    177  DHTEIDMVLMTPTA------------SMDDFTPSGEWDIVALPGRR---TVNPQDPS----YVDVTYDFIIKRKPLFYTI  237
γ     174  STNEIDLQLSQEDGqt----iewifiDPEAFTENGEWAIQHRPAKMLL---DPAAPAQEAgHQKVVFYLLIQRKPLFYVI  246
ε     172  NAEEVEFTFAVDNDgk---tinkidiDTEAYTENGEWAIDFCPGVIRR---HHGGATDGPgETDVIYSLIIRRKPLFYVI  245
δ     175  TAKEITLSLKQDAKenrtypvewiiiDPEGFTENGEWEIVHRPARVNV---DPRAPLDSPsRQDITFYLIIRRKPLFYII  251
```

```
α1    262  NVIIPCLLFSFLTGLVFYLPTDSG-EKMTLSISVLLSLTVFLLVIVELIPSTSSAVPLIGKYMLFTMVFVIASIIITVIV  340
α2    271  NLIIPCLLISCLTVLVFYLPSDCG-EKITLCISVLLSLTVFLLLITEIIPSTSLVIPLIGEYLLFTMIFVTLSIVITVFV  349
α3    247  NLIIPCLLISFLTVLVFYLPSDCG-EKVTLCISVLLSLTVFLLVITETIPSTSLVIPLIGEYLLFTMIFVTLSIVITVFV  325
α4    249  NLIIPCLLISCLTVLVFYLPSECG-EKITLCISVLLSLTVFLLLITEIIPSTSLVIPLIGEYLLFTMIFVTLSIVITVFV  327
α5    256  FLIIPCIGLSFLTVLVFYLPSNEG-EKICLCTSVLVSLTVFLLVIEEIIPSSSKVIPLIGEYLVFTMIFVTLSIMVTVFA  334
α6    246  NLIIPCLFISFLTVLVFYLPSDCG-EKVTLCISVLLSLTVFLLVITETIPSTSLVVPLVGEYLLFTMIFVTLSIVVTVFV  324
α7    265  NLLIPCVLISALALLVFLLPADSG-EKISLGITVLLSLTVFMLLVAEIMPATSDSVPLIAQYFASTMIIVGLSVVVTVIV  343
α9    243  NLLIPCVLISFLAPLSFYLPAASG-EKVSLGVTILLAMTVFQLMVAEIMPA-SENVPLIGKYYIATMALITASTALTIMV  320
α10   242  NLLLPCVLISLLAPLAFHLPADSG-EKVSLGVTVLLALTVFQLLLAESMPP-AESVPLIGKYYMATMTMVTFSTALTILI  319
β1    251  NVIAPCILITLLAIFVFYLPPDAG-EKMGLSIFALLTLTVFLLLLADKVPETSLSVPIIIKYLMFTMVLVTFSVILSVVV  329
β2    240  NLIIPCVLITSLAILVFYLPSDCG-EKMTLCISVLLALTVFLLLISKIVPPTSLDVPLVGKYLMFTMVLVTFSIVTSVCV  318
β3    239  FLIIPCLGLSFLTVLVFYLPSDEG-EKLSLSTSVLVSLTVFLLVIEEIIPSSSKVIPLIGEYLLFIMIFVTLSIIVTVFV  317
β4    238  NLIIPCVLTTLLAILVFYLPSDCG-EKMTLCISVLLALTFFLLLISKIVPPTSLDVPLIGKYLMFTMVLVTFSIVTSVCV  316
γ     247  NIIAPCVLISSVAILIHFLPAKAGgQKCTVAINVLLAQTVFLFLVAKKVPETSQAVPLISKYLTFLLVVTILIVVNAVVV  326
ε     246  NIIVPCVLISGLVLLAYFLPAQAGgQKCTVSINVLLAQTVFLFLIAQKIPETSLSVPLLGRFLIFVMVVATLIVMNCVIV  325
δ     252  NILVPCVLISFMVNLVFYLPADSG-EKTSVAISVLLAQSVFLLLISKRLPATSMAIPLIGKFLLFGMVLVTMVVVICVIV  330

α1    341  INTHHRSPSTHV-MPNWVRKVFIDTIPNIMFfstMKRPSREKQDKKIFTEDID------------ISDISGKPGP-----  402
α2    350  LNVHHRSPSTHT-MPHWVRGALLGCVPRWLL---MNRP------------------------------------------  383
α3    326  LNVHYRTPTTHT-MPSWVKTVFLNLLPRVMF---MTRPTSNEGNAQKPRPLYG-aELSNLNCFSRAESKGCKEGYPCQD-  399
α4    328  LNVHHRSPRTHT-MPTWVRRVFLDIVPRLLL---MKRPSVVKDNCRRLIESMHkmASAPRFWPEPEGEPPATSGTQSLH-  402
α5    335  INIHHRSSSTHNaMAPLVRKIFLHTLPKLLC---MRSHVDRYFT--------------------QKEETESGSGP-----  386
α6    325  LNIHYRTPTTHT-MPRWVKTVFLKLLPQVLL---MRWP------LDKTRGTGS-dAVPRGLARRPAKGKLASHGEPRHL-  392
α7    344  LQYHHHDPDGGK-MPKWTRVILLNWCAWFLR---MKRPGEDKVRPACQHKQRR--CSLASVEMSAVAPPPASNGNLLYIg  417
α9    321  MNIHFCGAEARP-VPHWARVVILKYMSRVLFvydVGESC-------LSPHHSR-eRDHLTKVYSKLPESNLKAARNKDLs  391
α10   320  MNLHYCGPSVRP-VPAWARALLLGHLARGLCvreRGEPCGQSRPPELSPSPQS-------PEGGAGPPAGPCHEPRCLC-  390
β1    330  LNLHHRSPHTHQ-MPLWVRQIFIHKLPLYLR---LKRPKPERDLMPEPPHCSS----PGSGWGRGTDEYFIRKPPSDFL-  400
β2    319  LNVHHRSPTTHT-MAPWVKVVFLEKLPALLF---MQQPRHHCARQRLRLRRRQ-rEREGAGALFFREAPGADSCT-----  388
β3    318  INVHHRSSSTYHpMAPWVKRLFLQKLPKLLC---MKDHVDRYSSPEKEESQPV----VKGKVLEKKKQKQLSDGE-----  385
β4    317  LNVHHRSPSTHT-MAPWVKRCFLHKLPTFLF---MKRPGPDSSPARAFPPSKScvTKPEATATSTSPSNFYGNSM-----  387
γ     327  LNVSLRSPHTHS-MARGVRKVFLRLLPQLLR---MHVRPLAPAAVQDTQSRLQ--NGSSGWSITTGEEVALCLPRSELL-  399
ε     326  LNVSQRTPTTHA-MSPRLRHVLLELLPRLLG---SPPPPEAPRAASPPRRASS------VGLLLRAEELILKKPRSELV-  394
δ     331  LNIHFRTPSTHV-LSEGVKKLFLETLPELLH---MSRPAEDGPSPGALVRRSS--SLGY---ISKAEEYFLLKSRSDLM-  400

α1    403  ------------------------------PPMGFHSPLIKHPEVKSAIE------------------------------  422
α2    384  ----------------------------------------------------pp--pvel-----------chp------  392
α3    400  ---gmcgychhrrikiSNFSANLTRSSSSESVDAVLSLSALSPEIKEAIQ------------------------------  446
α4    403  -------------------------PPSPSFCVPLDVPAEPGPSCKSPSDqlppqqpleaekasphpspgpcrpphgtqa  457
α5    387  --------------------------------------KSSRNTLEAALD------------------------------  398
α6    393  ---kecfhchk-----SNELATSKRRLSHQPLQWVVENSEHSPEVEDVIN------------------------------  434
α7    418  frgldgvhcvptpdsgVVCGRMACSPTHDEHLLHGGQPPEGDPDLAKILE------------------------------  467
α9    392  rk------------------------------------KDMNKRLKNDLG-cqgknpqeaes------------------  416
α10   391  -------------------------------------------RQEALLH------------------------------  397
β1    401  ---fp---------kpNRFQPELSAPDLRRFIDGPNRAVALLPELREVVS------------------------------  438
β2    389  --------cfvnrasvQGLAGAFGAEPA-PVAGPGRSGEPCGCGLREAVD------------------------------  429
β3    386  --------------------------------------KVLVAFLEKAAD------------------------------  397
β4    388  --------yfvnpasaASKSPAGSTPVAIPRDFWLRSSGRFRQDVQEALE------------------------------  429
γ     400  ---fqq---wqrqglvAAALEKLEKGPELGLSQFCGSLKQAAPAIQACVE------------------------------  443
ε     395  ---feg----------------QRHRQGTWTAAFCQSLGAAAPEVRCCVD------------------------------  425
δ     401  ---fek---qserhglARRLTTARRPP----ASSEQAQQELFNELKPAVD------------------------------  440

α1         --------------------------------------------------------------------------------
α2    393  --lrlklspsyhwlesnvdaeerevvveeedrwacagh--vap-----svgtlcshghlhsg------------------  445
α3         --------------------------------------------------------------------------------
α4    458  pglakarslsvqhmsspgeaveggvrcrsrsiqycvprddaapeadgqaagalasr-nthsaelpppdqpspckctckke  536
α5         --------------------------------------------------------------------------------
α6         --------------------------------------------------------------------------------
α7         --------------------------------------------------------------------------------
α9    417  ---------------------------------ycaqyk-------------vltr------------------------  426
α10        --------------------------------------------------------------------------------
β1         --------------------------------------------------------------------------------
β2         --------------------------------------------------------------------------------
β3         --------------------------------------------------------------------------------
β4         --------------------------------------------------------------------------------
γ          --------------------------------------------------------------------------------
ε          --------------------------------------------------------------------------------
δ          --------------------------------------------------------------------------------
```

```
α1    423  ---------------------------------GIKYIAETMKSDQESNNAAAEWKYVAMVMDHILLGVFMLVCIIGTLA  469
α2    446  --asgpkaeallqege-----lllsphmqkaleGVHYIADHLRSEDADSSVKEDWKYVAMVIDRIFLWLFIIVCFLGTIG  518
α3    447  ---------------------------------SVKYIAENMKAQNEAKEIQDDWKYVAMVIDRIFLWVFTLVCILGTAG  493
α4    537  pssvspsatvktrstkappphlplspaltraveGVQYIADHLKAEDTDFSVKEDWKYVAMVIDRIFLWMFIIVCLLGTVG  616
α5    399  ---------------------------------SIRYITRHIMKENDVREVVEDWKFIAQVLDRMFLWTFLFVSIVGSLG  445
α6    435  ---------------------------------SVQFIAENMKSHNETKEVEDDWKYVAMVVDRVFLWVFIIVCVFGTAG  481
α7    468  ---------------------------------EVRYIANRFRCQDESEAVCSEWKFAACVVDRLCLMAFSVFTIICTIG  514
α9    427  ---------------------------------NIEYIAKCLKDHKATNSKGSEWKKVAKVIDRFFMWIFFIMVFVMTIL  473
α10   398  ---------------------------------HVATIANTFRSHRAAQRCHEDWKRLARVMDRFFLAIFFSMALVMSLL  444
β1    439  ---------------------------------SISYIARQLQEQEDHDALKEDWQFVAMVVDRLFLWTFIIFTSVGTLV  485
β2    430  ---------------------------------GVRFIADHMRSEDDDQSVSEDWKYVAMVIDRLFLWIFVFVCVFGTIG  476
β3    398  ---------------------------------SIRYISRHVKKEHFISQVVQDWKFVAQVLDRIFLWLFLIVSVTGSVL  444
β4    430  ---------------------------------GVSFIAQHMKNDDEDQSVVEDWKYVAMVVDRLFLWVFMFVCVLGTVG  476
γ     444  ---------------------------------ACNLIACARHQQSHFDNGNEEWFLVGRVLDRVCFLAMLSLFICGTAG  490
ε     426  ---------------------------------AVNFVAESTRDQEATGEEVSDWVRMGNALDNICFWAALVLFSVGSSL  472
δ     441  ---------------------------------GANFIVNHMRDQNNYNEEKDSWNRVARTVDRLCLFVVTPVMVVGTAW  487

α1    470  VFAGRLIELNQQG-------------------------------------------------------------------  482
α2    519  LFLPPFLAGMI---------------------------------------------------------------------  529
α3    494  LFLQPLMAREDA--------------------------------------------------------------------  505
α4    617  LFLPPWLAGMI---------------------------------------------------------------------  627
α5    446  LFVPVIYKWANIL---Ipvhignank------------------------------------------------------  468
α6    482  LFLQPLLGNTGKS-------------------------------------------------------------------  494
α7    515  ILMSAPNFVEAVSkdfA---------------------------------------------------------------  531
α9    474  IIARAD--------------------------------------------------------------------------  479
α10   445  VLVQAL--------------------------------------------------------------------------  450
β1    486  IFLDATYHLPPPDpf----------p------------------------------------------------------  501
β2    477  MFLQPLFQNYTTTtflHsdhsapssk------------------------------------------------------  502
β3    445  IFTPALKMW---L---Hsyh------------------------------------------------------------  458
β4    477  LFLPPLFQTHAAS---Egpyaaqrd-------------------------------------------------------  498
γ     491  IFLMAHYNRVPALpfpGdprpy----------------------------------------------------------  512
ε     473  IFLGAYFNRVPDLpyaPciqp-----------------------------------------------------------  493
δ     488  IFLQGVYNQPPPQpfpGdpysynvqdkrfihttpswwwncbinlmnihgovtoolscobaltcobaltcgicmdgetcob  567

α1         ------------
α2         ------------
α3         ------------
α4         ------------
α5         ------------
α6         ------------
α7         ------------
α9         ------------
α10        ------------
β1         ------------
β2         ------------
β3         ------------
β4         ------------
γ     513  -lpspd------  517
ε          ------------
δ     568  altridvbtjjk  579
```

Figure S2. Negative isotype control staining. Representative micrographs evaluating non-specific staining of the bronchial epithelia on FFPE tissues using anti-isotype matched GFP IgG antibodies (in red) from mouse (up) or rabbit (down), and DAPI (cell nuclei, blue). Magnification corresponding to the selected area is shown.

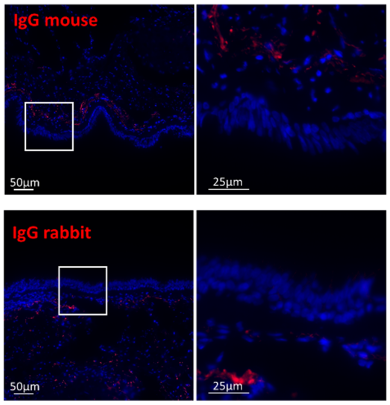

Figure S3. Localization of nAChRs on lung tissues from the Human Protein Atlas. Representative micrographs showing the bronchial epithelia on FFPE lung tissues: immunohistochemistry for $\alpha 3$, $\alpha 7$, and $\beta 1$.

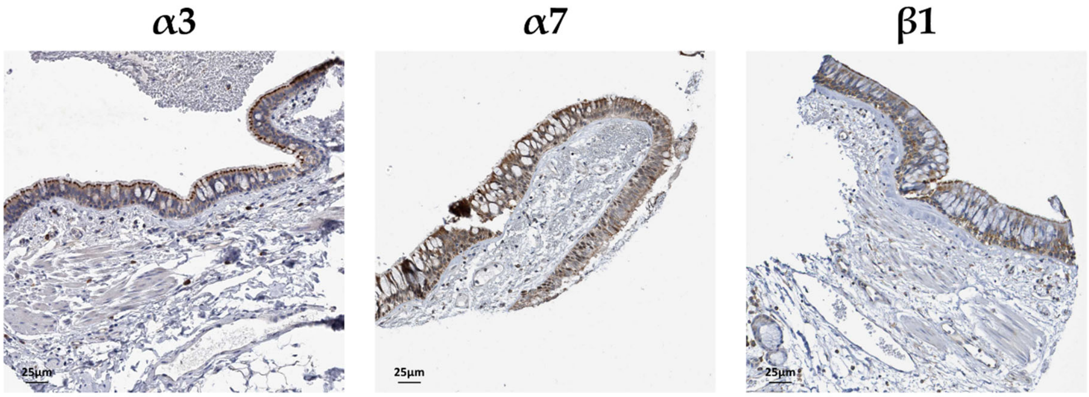

Supplement: Supplementary file 1 [file ijms-21-07446-s001.pdf]
